# Vibration Viscosity Sensor for Engine Oil Monitoring Using Metal Matrix Piezoelectric Composite

**DOI:** 10.3390/ma12203415

**Published:** 2019-10-18

**Authors:** Tetsuro Yanaseko, Hiroshi Sato, Isao Kuboki, Karla Mossi, Hiroshi Asanuma

**Affiliations:** 1 Department of Mechanical Engineering, Kogakuin University, 2665-1, Nakano-cho, Hachioji-shi, Tokyo 192-0015, Japan; i.kuboki@cc.kogakuin.ac.jp; 2 Advanced Manufacturing Research Institute, National Institute of Advanced Industrial Science and Technology, 1-2-1, Namiki, Tsukuba-shi, Ibaraki 302-8564, Japan; h-sato@aist.go.jp; 3 Mechanical and Nuclear Engineering Department, Virginia Commonwealth University, 401 West Main Street, PO Box 843015, Richmond, VA 23284-3015, USA; kmmossi@vcu.edu; 4 Department of Mechanical Engineering, Chiba University, 1-33, Yayoi-cho, Inage-ku, Chiba-shi, Chiba 263-8522, Japan; asanuma@faculty.chiba-u.jp

**Keywords:** viscosity, smart materials, piezoelectric composite, metal matrix composite, active sensor

## Abstract

Lubricants such as engine oil play an important role in preventing machine wear and damage. Monitoring the deterioration of lubricating oils is a significant technical issue in machine maintenance. In this study, a sensor for monitoring engine oil viscosity was developed using a metal-core piezoelectric fiber/aluminum composite. This composite is a piezoelectric ceramic that is reinforced by a metal matrix; it is expected to be utilized in harsh environments such as the inside of an engine. An active type measurement method was employed to monitor variations in the viscosity of glycerin solution as a model liquid. In this method, a self-generated vibration is correlated to the viscosity of a liquid by measuring the damped vibration amplitude and the variation in the resonance frequency. The results showed that the vibration had a high sensitivity to the liquid viscosity; further, it was observed that the shift in resonance frequency correlated to a wider range of measurable viscosity. Both measured parameters indicate that the metal-core piezoelectric fiber/aluminum composite is a viable sensor for engine oil monitoring.

## 1. Introduction

In recent years, the requirement for engine oil performance has increased with the improved performance of engines and better operating conditions [1,2]. Engine oil protects the engine from any energy losses due to the friction generated from parts in the engine, heat generation, and friction on the contact surface [3,4]. In terms of operating conditions, engine oil differs significantly from industrial lubricating oil because it is strongly influenced by external contaminants [5]. Engine oil is used under high temperature and high pressure in an environment contaminated by sludge; this environment comprises of carbon as an incomplete combustion product of the engine oil itself, and fuel gas, as well as debris, entering from gaps of the air filter and the part under friction. It is considered that the deterioration of oil is promoted by the mixing of these sludge components. Oxidation naturally occurs with usage, and the temperature of the oil subsequently increases. When the oil loses its ability to detoxify the contaminants and prevent its own deterioration, an oil change is required. This quality and the time when the engine oil should be changed is difficult to judge, except via general inspection [6] of the engine oil. This is because engine oil deteriorates according to the different wear resistances of engine parts owing to their difference in structure or material. Therefore, it is difficult to judge that engine oil has deteriorated under the same engine oil and same operating conditions [7]. These factors hinder the prediction of engine oil degradation and render its detection more difficult and complex.

Various methods have been developed to rapidly analyze and detect the deterioration of lubricating oils such as engine oils on site in recent years. For example, oil degradation was evaluated by measuring the relative dielectric constant of oil. This degradation increases with the disappearance of molecularly broken additives in the oil [8,9,10]; further, it increases within the passing rate or absorption rate of infrared rays [11,12,13] and the increase in the viscosity of the oil [14,15,16]. Although these methods have merits and demerits, the engine oil measurement system should be robust and compatible with the harsh environment of the engine; it should also be compact for easy mounting on the vehicle. Further, these methods are compared, and it is observed that the vibration type sensor size is small, which is advantageous in mountability.

There are two popular methods for viscosity measurement. One involves measuring the time for which a liquid passes through a fixed capillary-type flow path to obtain the dynamic viscosity [17], and the other involves measuring the rotational torque generated in the liquid, such as the Brookfield type [18]. However, in recent years, the demand for measuring and managing viscosity data online is increasing for ease and accuracy. There is also an increasing demand for measuring the viscosity as an inherent characteristic of the liquid in the state of minimal interference with the measurement system; this measurement is calculated without applying a large energy load such as a center of gravity movement or rotational force on the liquid to be measured. In order to meet these requirements, the vibrational viscosity measurement has been proposed as a new measurement method [19].

In this study, a vibration viscosity measuring sensor capable of measuring the viscosity of engine oil was used for monitoring the deterioration of the oil using a metal-core piezoelectric fiber/aluminum composite [20,21]. This composite embeds a metal-core piezoelectric fiber [22,23] in the aluminum matrix. Consequently, the strength and durability of the piezoelectric ceramics are improved; moreover, it has a high resistance to external noise, and can be used in harsh environments [24]. There are some studies of measuring viscosity with vibrators using piezoelectric materials [25,26]. Conventionally, it is necessary that a process is known for fabricating relatively complicatedly shaped electrodes on the surface of piezoelectric ceramics for the purpose of using ceramics simultaneously as sensors and actuators. However, in this composite, the electrode formation process is achieved by embedding the fibers; thus, the electrode formation process is unnecessary. In addition, since the piezoelectric ceramics are exposed to the measurement liquid, it is expected that these will be easily damaged in a harsh environment. An active sensor is in development that overcomes these problems by using a composite in which two piezoelectric fibers are embedded in aluminum. Since the two fibers can be used independently, the sensor uses one fiber for vibrational excitation, and another for measuring the value of the vibration depending on the viscosity of the liquid, such as the amplitude, the resonance frequency. Using this method, it is possible to monitor oil viscosity of an engine in situ. A glycerin aqueous solution was used as the viscosity measurement liquid in this study. This is because the maximum and minimum viscosities defined by the current engine oil standard SAE-J300 [27] can be achieved by changing the concentration of glycerin [28] such that each grade of oil will not be required.

## 2. Materials and Methods

### 2.1. Viscosity Sensor Fabrication

**Figure 1:**
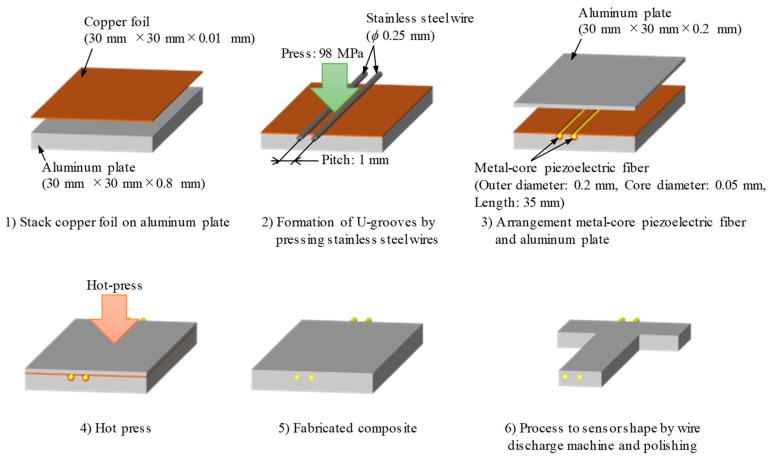
Schematic of fabrication procedure of viscosity sensor. It is possible to embed a piezoelectric fiber in aluminum without damage by interphase forming/bonding method.

Figure 1 shows the schematic of the fabrication procedure of the viscosity sensor. First, 0.2 mm and 0.8 mm thick aluminum plates and 0.01 mm thick copper foil were cut to a length of 30 mm and width of 30 mm. This was followed by sanding with #600 water-resistant abrasive paper to remove the oxide film and cause degreasing by acetone. The copper foil is stacked on the top of the 0.8 mm thick aluminum plate. Two U-grooves with a pitch of 1 mm were formed by pressing stainless steel wires (SUS304, diameter: 0.25 mm) to the copper foil and aluminum plate with a pressure of 98 MPa under holding time of 0.18 ks. The metal-core piezoelectric fibers (Outer diameter: 0.2 mm. core diameter: 0.05 mm, length: 35 mm, properties are shown in Table 1 [29]) were arranged in the formed U-groove, an aluminum plate of thickness 0.2 mm was stacked on them. Subsequently, the piezoelectric fibers were embedded in the aluminum plate by hot pressing at a temperature of 873 K, pressure of 2.2 MPa, holding time of 2.4 ks, and degree of vacuum of 0.1 kPa. By using copper as the insert material, eutectic reaction between copper and aluminum occurs during hot pressing; only the fiber periphery becomes the liquid phase. The resulting liquid phase reduces the pressure on the piezoelectric ceramics during the process and prevents the piezoelectric ceramics from being fractured. Subsequently, as the diffusion of copper in aluminum progresses, the liquid phase is isothermally solidified; further, the composite of the piezoelectric fiber in aluminum disappears. This method is called interphase forming/bonding (IF/B) method, and it is a method developed for compounding fragile functional materials in metal [30].

**Figure 2:**
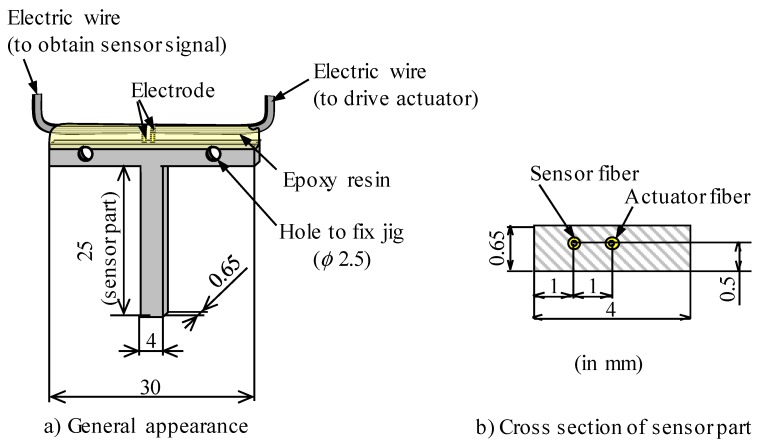
Schematic of fabricated sensor, (**a**) general appearance and (**b**) cross-section of sensor part. The fiber is eccentric with respect to the cross section and bending vibration of the cantilever occurs due to the expansion and contraction of the fiber.

After hot pressing, the specimen was processed using a wire discharge machine and polished to the shape shown in Figure 2. The platinum wires were exposed by removing the piezoelectric ceramic layer from the fiber that protruded from the end of the specimen. Subsequently, the exposed platinum wire and copper foil were attached to each other by silver paste (DOTITE 510, Fujikura Kasei Co., Ltd., Tokyo, Japan), and electrodes were coated with an epoxy resin (# 16051, Konishi Co., Ltd., Osaka-shi, Japan). Each fiber was poled by applying a DC voltage of 300 V for 1.8 ks between the electrode and aluminum matrix by a high-voltage power supply (A100603, Kepco, Inc., New York City, NY, USA.

**Figure 3:**
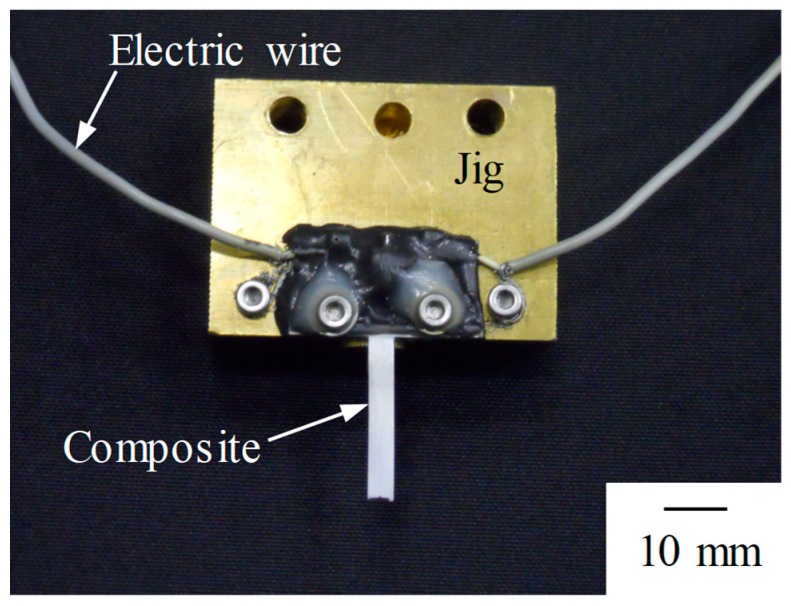
Photograph of fabricated sensor and jig. The sensor is fixed to the brass jig.

Figure 3 shows the appearance of the prepared sensor. This sensor is designed to generate bending vibrations when the fiber is expanded or contracted by offsetting the fiber 0.1 mm from the center in the thickness direction.

### 2.2. Vibration Characterization of the Sensor

**Figure 4:**
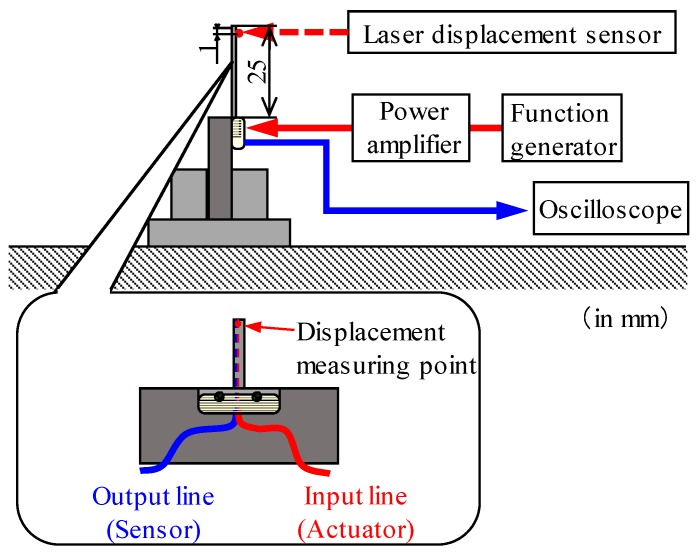
Schematic of test system to evaluate the vibration characteristics of the sensor in the air. The vibration characteristics were evaluated by measuring the displacement at point of 1 mm from the tip of the cantilever.

Using the piezoelectric fiber at the center of the sensor as the actuator and the other as the detector, it was confirmed that the specimen functions as an actuator as well as a sensor. Figure 4 shows the test system. The sensor was fixed by the jig and vibrated by the piezoelectric fiber at the center of the sensor by using a function generator (WF1944, NF Corp., Yokohama-shi, Japan) and a power amplifier (HSA4051, NF Corp., Yokohama-shi, Japan). The output voltage generated from the other piezoelectric fiber was measured using an oscilloscope (DL1740, Yokogawa Electric Corporation, Musashino-shi, Japan). The displacement of the sensor under the driving voltage measured using a laser displacement meter (LC-2450, Keyence Corp., Osaka-shi, Japan) was positioned 1 mm from the tip of the sensor. The voltage applied to the piezoelectric fiber placed at the center was 15 V at a range of 810 to 830 Hz in the air.

### 2.3. Active Viscosity Measurement

First, active viscosity measurement was conducted using two embedded piezoelectric fibers. The fiber placed at the center of the sensor vibrated the sensor, while the other generated the output voltage. Using this method, the viscosity can be measured via the relationship between the resonance frequency or maximum output voltage of the sensor and viscosity.

The vibration of the sensor at this time is a damped forced vibration, because it occurs in the liquid. The equation of motion is as follows [31],

(1) $$m\ddot{x}+c\dot{x}+kx=F\sin\frac{f}{2\pi }t$$

where, *m* is the mass, *c* is the damping coefficient, *k* is the elastic constant, *F* is the amplitude of the applied force, *f* is the frequency, and *t* is the time. The damped resonance frequency *f*_d_ is given by Equation (2) [31] and the amplitude ratio *M* is given by Equation (3) [31].

(2) $$f_{d}=\frac{1}{2\pi }\sqrt{\frac{k}{m}}\sqrt{1-2\zeta^{2}}$$

(3) $$M=\frac{1}{\sqrt{{\left(1-{\left(f/f_{n}\right)}^{2}\right)}^{2}+4\zeta^{2}{\left(f/f_{n}\right)}^{2}}}$$

Here,

(4) $$f_{n}=\frac{1}{2\pi }\sqrt{\frac{k}{m}}$$

(5) $$\zeta =\frac{c}{2\sqrt{mk}}$$

when considering the viscous drag, the drag consists of two components; one is viscous resistance inherent to the vibration system, the other is the viscous drag that the sensor receives from the liquid [32]. Therefore,

(6) $$c=R_{m}+A\sqrt{\pi f\rho \mu }$$

where, *A* is the sensor area, *ρ* is the density of liquid and *μ* is the viscosity of liquid.

Substituting Equation (6) into Equations (2) and (3), the resonance frequency and amplitude ratio are as follows.

(7) $$f_{d}=\frac{1}{2\pi }\sqrt{\frac{k}{m}}\sqrt{1-\frac{{\left(R_{m}+A\sqrt{\pi f\rho \mu }\right)}^{2}}{2mk}}$$

(8) $$M=\frac{1}{\sqrt{{\left(1-{\left(f/f_{n}\right)}^{2}\right)}^{2}+\frac{{\left(R_{m}+A\sqrt{\pi f\rho \mu }\right)}^{2}}{mk}{\left(f/f_{n}\right)}^{2}}}$$

Thus, the physical quantity that can be measured in this method is the “static viscosity”, which is the product of density *ρ* and viscosity *μ*. In addition, the driving frequency is equal to the resonance frequency *f_d_* (*f* = *f_d_*) because the value at resonance is used in the measurement of the active measurement; therefore, Equation (8) becomes,

(9) $$M=\frac{1}{2\zeta \sqrt{1-\zeta^{2}}}=\frac{1}{\frac{R_{m}+A\sqrt{\pi f_{d}\rho \mu }}{\sqrt{mk}}\sqrt{1-\frac{{\left(R_{m}+A\sqrt{\pi f_{d}\rho \mu }\right)}^{2}}{2mk}}}$$

It is evident that the resonance frequency and amplitude ratio as described depend on the static viscosity, and it would be possible to measure the static viscosity by determining these parameters. It is noteworthy that the measurable range is limited to critical damping or less.

**Figure 5:**
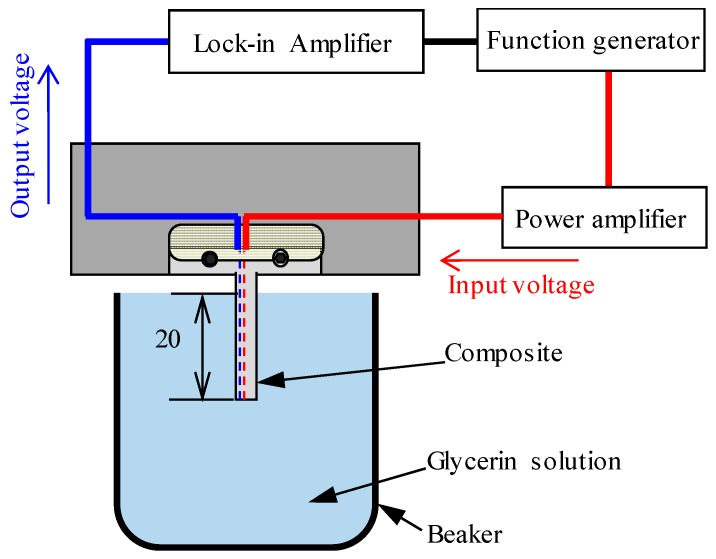
Schematic of active style viscosity measurement system. The effect of changes in static viscosity on resonance frequency and output voltage was evaluated using this system.

The test system is shown in Figure 5. The sample was fixed such that a 20 mm part from the tip was immersed in the aqueous solution.

The drive-side piezoelectric fiber placed in the center was connected to the function generator through the power amplifier. The applied voltage was maintained constant at 10.2 V, and the frequency was changed continuously from 350 Hz to 550 Hz to produce vibrations in the sensor.

At that juncture, the output voltage of the piezoelectric fiber on the detection side was measured by a lock-in amplifier. The frequency at which the output voltage was the highest was considered the resonance frequency, and its relationship with the viscosity of the aqueous solution was determined.

**Figure 6:**
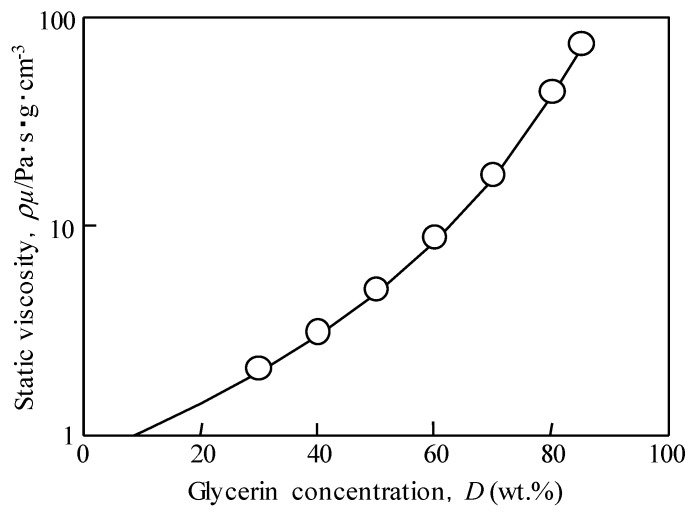
Effect of glycerin concentration of glycerin solution on the static viscosity [28]. Viscosity of several grades of engine oil can be reproduced by changing the concentration.

Pure water and an aqueous solution of glycerin were used as model solutions for viscosity measurement. The relationship between the concentration and static viscosity of an aqueous glycerol solution is shown in Figure 6 [28], in which the concentration of the aqueous glycerol solution *D* to be measured was adjusted to 30%, 40%, 50%, 60%, 70%, 80%, and 85%. The static viscosity at these concentrations was used as a model solution to meet the minimum and maximum values (minimum: less than 0.95 Pa·s·g·cm^−3^, maximum: 51 Pa·s·g·cm^−3^) of engine oil standard SAE-J300 [27]. The static viscosity of glycerin aqueous solution is 0.01 (*D* = 0, pure water) to about 93 Pa·s·g/cm^3^ (*D* = 85%); this range is in accordance with the viscosity range of engine oil that specified by the standard. The standard SAE-J300 specifies kinematic viscosity and viscosity of each grade of oils, therefore, the static viscosity is calculated from these values and their densities [33].

After the viscosity measurement, cross-sectional observation was performed to evaluate the microstructure of the sensor material. In the observation, the center of the cantilever part of the sensor was cut in the direction perpendicular to the piezoelectric fiber; the observation was performed with a scanning electron microscope (SEM).

## 3. Results and Discussion

### 3.1. Evaluation of Vibration Characteristics of the Sensor

**Figure 7:**
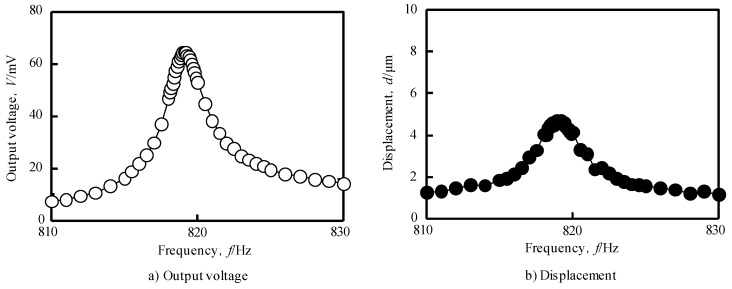
Effect of frequency on (**a**) output voltage and (**b**) displacement of sensor in vibration test in the air. The peak position of output voltage (819.1 Hz) and the peak position of displacement (819.2 Hz) are in good agreement.

Figure 7a,b show the influence of the driving frequency on the displacement of the sample and the output voltage.

The output voltage generated by the sensor is maximum at 64.6 mV when the frequency is 819.1 Hz, and the displacement of the sample is maximum at 4.6 m when the frequency is 819.2 Hz; both frequencies are in good agreement.

Therefore, it is understood that there is a correlation between the displacement of the sensor due to the vibration generated by one of the composited fibers and the output voltage generated from the other fiber. This result is based on the fact that the output voltage obtained from the metal-core piezoelectric fiber is proportional to the given strain, as clarified in the previous studies [20,24], thus, this sensor can measure the strain owing its vibration. Here, as the sensor is cantilever-shaped, the first-order resonance frequency can be obtained by the following equation [31],

(10) $$f_{n}=\frac{1}{2\pi }{\left(\frac{1.875}{l}\right)}^{2}\sqrt{\frac{EI}{\rho_{c}A_{c}}}$$

where *l* is the length of the cantilever, *E* is Young’s modulus, *I* is the moment of inertia of area, *ρ_c_* is the density of cantilever material, and *A*_c_ is the area of cross-section of the cantilever. Substituting each value for the fabricated sensor showed as Table 2 into Equation (10), it was found that the resonance frequency at free vibration of the sensor in the air is 828 Hz. This value is very close to the frequency of 819 Hz when the maximum voltage occurs in the experiment. Thus, it is clear that this frequency is the primary resonance frequency in the air of this sensor, and the resonance of this sensor can be measured by measuring the output voltage generated from this sensor. Hence, it was possible to identify the frequency.

### 3.2. Viscosity Measurement by Active Sensor Style

**Figure 8:**
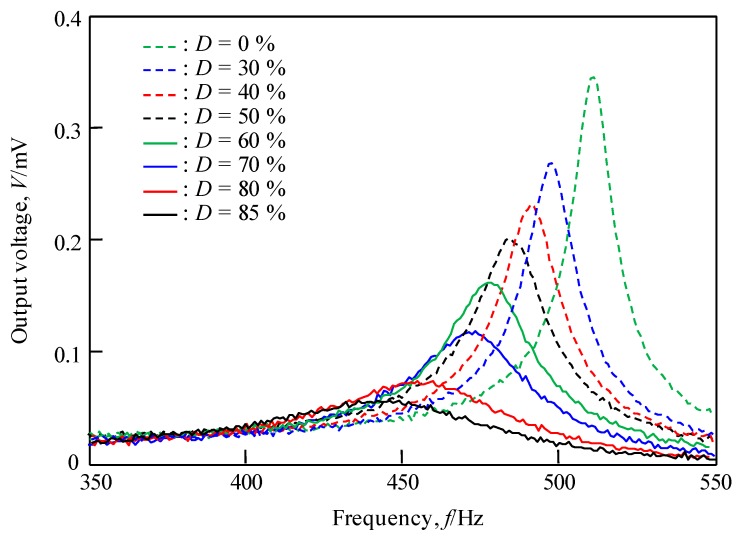
Relationship between frequency and output voltage when changing the concentration of glycerin solution *D* from 0% to 85%. As the concentration increases, the peak height decreases and the width increases.

From the output voltage measurements of the sensor for the glycerin aqueous solutions of various viscosities, the resonance frequency was confirmed in the same manner as discussed above. Figure 8 shows the results of the active viscosity measurement when the concentration of aqueous glycerin solution was varied from 0% to 85%.

From the figure, it can be observed that the output voltage obtained from this sensor decreases as the concentration of the solution to be measured increases. Further, it can be seen that the frequency for the maximum output voltage is generated shifts at the same time. It is also clear that the critical attenuation has not been reached because a clear peak is seen for the frequency.

**Figure 9:**
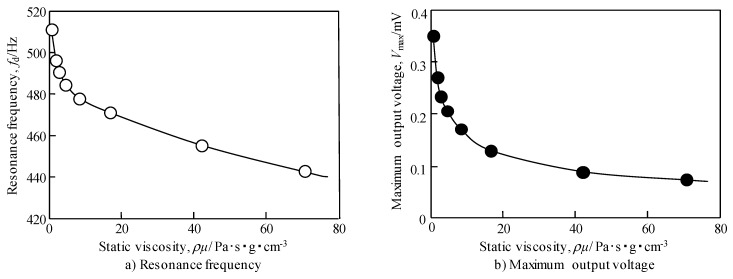
Effect of static viscosity on (**a**) resonance frequency and (**b**) maximum output voltage by measuring active style. In the high viscosity region, the change in output voltage becomes gradual.

Figure 9 shows the relationship between the static viscosity of the aqueous solution of glycerin and resonance frequency and the maximum output voltage of the sensor. From the figure, it can be seen that the resonant frequency and maximum output voltage of the sensor decrease and monotonically decrease as the static viscosity of the solution increases. The amount of change is larger in the low static viscosity region and decreases as the static viscosity increases. Comparison of the two results shows that the change in resonant frequency is small (Resonance frequency change: 516 Hz to 445 Hz, 13.7% change, output voltage change: 0.36 mV to 0.075 mV, 79.2% change).

**Figure 10:**
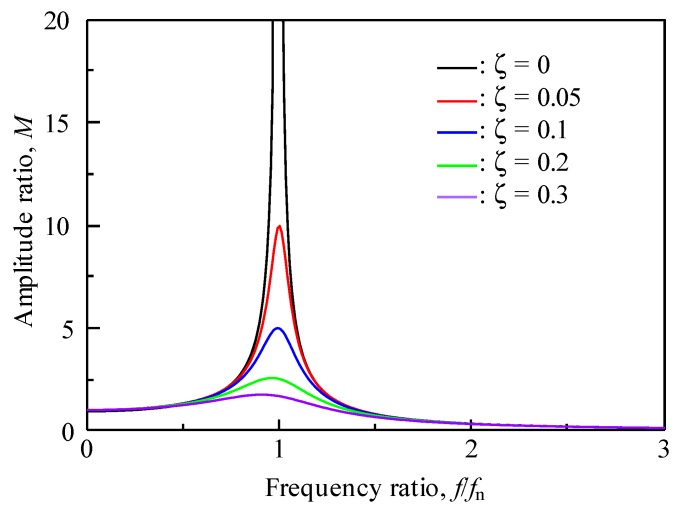
Effect of frequency ratio on amplitude ratio based on Equation (3). (The amplitude ratio diverges infinitely when *ζ* = 0). As the damping factor increases, the resonance frequency and amplitude decrease.

**Figure 11:**
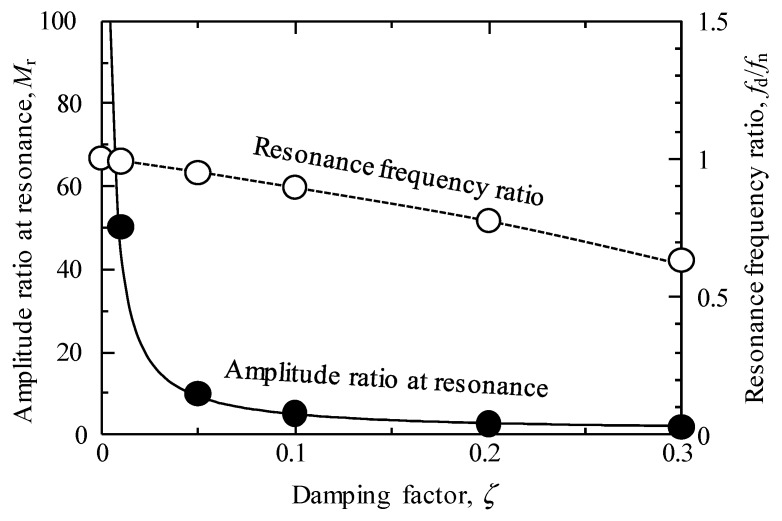
Effect of damping factor on amplitude ratio at resonance and resonance frequency ratio *f_d_*/*f_n_* based on Equation (3). The amplitude ratio decreases rapidly with increasing damping factor (*ζ* < 0.05) and does not change much thereafter. On the other hand, the change of the resonance frequency gradually decreases as the damping factor increases.

This relationship is clear when comparing Equations (2) and (3). This is because the term that the static viscosity effects in Equation (2) is $\sqrt{1-\zeta^{2}}$, where in Equation (3) it is $1/2\zeta \sqrt{1-\zeta^{2}}$. Figure 10 shows the effect of the frequency ratio *f*/*f_n_* on amplitude ratio based on Equation (3), and Figure 11 shows the effect of the damping factor *ζ* on the resonance frequency ratio *f_d_*/*f_n_* and amplitude ratio at resonance *M*_r_ from the results shown in Figure 10.

From these figures, it can be seen that in amplitude measurements using Equation (3), the changes are extremely sensitive when *ζ* is small; i.e., the sensitivity is high. In the measurement within the region where the *ζ* is large, i.e., in the high-viscosity region, the amplitude ratio hardly changes; this indicates that the measurement by output voltage is not suitable for the high-viscosity region. Conversely, the change in resonance frequency is mild, which indicates that it is suitable for the measurement in a wide viscosity range. Moreover, in Equation (6), the mechanical attenuation component that is unique to the sensor and the area of the sensor vibration of both the surfaces affect the damping coefficient; therefore, it is expected that the sensitivity can be changed by designing the sensor shape to change these value. In addition, it is important to improve the piezoelectric performance of this composite in order to improve the sensitivity as a viscosity measurement sensor. This is because the improvement of the piezoelectric performance provides a larger vibration amplitude and signal level. The microstructure improvement of this composite is effective for improving piezoelectric performance.

**Figure 12:**
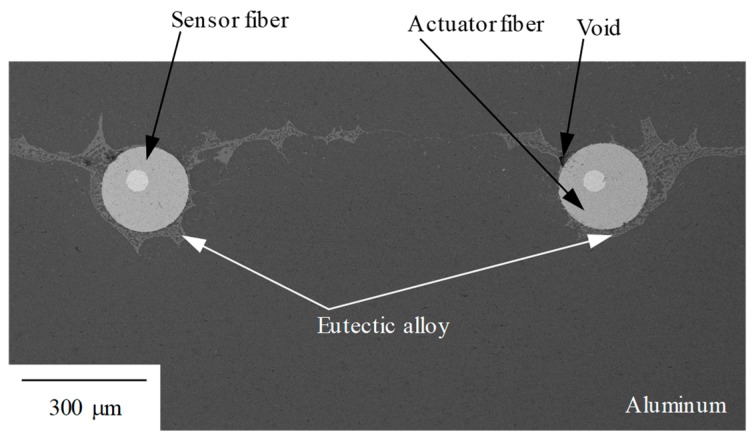
Backscattered electron composition image of cross section of cantilever part of fabricated viscosity sensor. Eutectic alloys and voids cause the sensitivity of the sensor to decrease [34].

Figure 12 shows the image of the microstructure of the sensor observed by SEM. It can be seen that the two fibers can be embedded in the aluminum matrix without cracking. However, the voids and the retention of eutectic alloy that formed by the IF/B method were confirmed, and the previous study has shown that void and eutectic alloy residue reduce the piezoelectric performance of the composite [34].

This is because there is no contact between the matrix and the piezoelectric ceramic at the place where the void is present, so no stress is generated in the piezoelectric ceramic at that portion, and the output is reduced as a result. Even in the case of the eutectic alloy remain, the eutectic alloy has a high Young’s modulus (the eutectic alloy is a mixture of α phase which is the solid solution of aluminum and CuAl_2_ that has Young’s modulus 110 GPa [35]); therefore, stress transfer between matrix and piezoelectric ceramics Is inhibited, and the output decreases.

Thus, viscosity measurement with this sensor is possible at any resonance frequency and maximum output voltage. It was, therefore, suggested that using the resonance frequency might be suitable for measuring a wide range of static viscosity, and high accuracy measurement is possible in the low static viscosity range using the maximum output voltage. In addition, viscosity measurement using the change of resonance frequency can be measured even by natural vibration excited by external energy. Examples include the impact that occurs when the vehicle drives, thus, it should be expected to be applied to passive viscosity measurement.

## 4. Conclusions

The following findings were obtained from the investigation of the viscosity sensor for engine oil using metal-core piezoelectric fiber/aluminum composite and evaluation of viscosity measurement characteristics.

(1) The sensor device fabricated in this study can monitor the vibration generated by the drive side fiber with the detection side fiber, and the sensor’s resonant frequency can be identified by the maximum value of the output voltage.

(2) The resonance frequency and maximum output voltage of this sensor monotonously decrease with increasing viscosity of the glycerin solution; thus, this device can be used as a viscosity sensor. Moreover, it was found from the analysis of the equation of motion that the method using the shift of resonance frequency is suitable for the measurement in the high viscosity region and that using the change in output voltage is suitable for the measurement in the low viscosity region.

(3) The sensor sensitivity depends on the area and microstructure of the sensor; therefore, it is suggested that the sensitivity can be improved by optimizing these parameters.

## Figures and Tables

**Table 1:** Properties of metal-core piezoelectric fiber [29].

Young’s Modulus/GPa	Density/g·cm^−3^	Electromechanical Coupling Coefficient, *K*_p_	Piezoelectric Constant, *d*_33_/pm·V^−1^	Curie Temperature/K
30–50	7.7	0.68	480	558

**Table 2:** Composite dimension and physical properties to calculate resonance frequency.

Length, *l*/m	Width, *b*/m	Thickness, *h*/m	Young’s Modulus, *E*/GPa	Density, *ρ_c_*/g·cm^−3^
0.0256	0.004	0.00065	69	2.7
